# Why rural women do not use primary health centres for pregnancy care: evidence from a qualitative study in Nigeria

**DOI:** 10.1186/s12884-019-2433-1

**Published:** 2019-08-05

**Authors:** Lorretta Favour C. Ntoimo, Friday E. Okonofua, Brian Igboin, Chioma Ekwo, Wilson Imongan, Sanni Yaya

**Affiliations:** 1 Women’s Health and Action Research Centre, Km 11 Benin-Lagos Expressway, Benin City, Edo State Nigeria; 2 The Federal University, Oye-Ekiti, Ekiti State Nigeria; 3 The University of Medical Sciences, Ondo City, Ondo State Nigeria; 4 The Centre of Excellence in Reproductive Health Innovation (CERHI), University of Benin, Benin City, Nigeria; 5 The University of Ottawa, Ottawa, Canada

**Keywords:** Maternal deaths, Pregnant women, Rural communities, Edo state, Nigeria

## Abstract

**Background:**

While Primary Health Care has been designed to provide universal access to skilled pregnancy care for the prevention of maternal deaths in Nigeria, available evidence suggests that pregnant women in rural communities often do not use Primary Health Care Centres for skilled care. The objective of this study was to investigate the reasons why women do not use PHC for skilled pregnancy care in rural Nigeria.

**Methods:**

Qualitative data were obtained from twenty focus group discussions conducted with women and men in marital union to elicit their perceptions about utilisation of maternal and child health care services in PHC centres. Groups were constituted along the focus of sex and age. The group discussions were tape-recorded, transcribed verbatim and analyzed thematically.

**Results:**

The four broad categories of reasons for non-use identified in the study were: 1) accessibility factors – poor roads, difficulty with transportation, long distances, and facility not always open; 2) perceptions relating to poor quality of care, including inadequate drugs and consumables, abusive care by health providers, providers not in sufficient numbers and not always available in the facilities, long waiting times, and inappropriate referrals; 3) high costs of services, which include the inability to pay for services even when costs are not excessive, and the introduction of informal payments by staff; and 4) Other comprising partner support and misinterpretation of signs of pregnancy complications.

**Conclusion:**

Addressing these factors through adequate budgetary provisions, programs to reduce out-of-pocket expenses for maternal health, adequate staffing and training, innovative methods of transportation and male involvement are critical in efforts to improve rural women’s access to skilled pregnancy care in primary health care centres in the country.

## Background

Several reports indicate higher rates of maternal mortality in rural parts of Nigeria as compared to urban areas [1–4]. The Federal Government of Nigeria has adopted primary health care (PHC) as a policy to achieve universal health coverage for citizens and to ensure that women, especially in rural areas, gain access to evidence-based skilled pregnancy care for the prevention of maternal morbidity and mortality [5]. In consonance with this policy, the Federal Ministry of Health launched the Primary Health Care Under One Roof programme in 2014 for the purpose of revitalizing primary health care infrastructure to deliver effective and efficient services to vulnerable populations [6].

Despite these efforts, available reports indicate substantial under-utilisation of PHC centres for skilled pregnancy care by vulnerable rural women throughout the country [7–10]. With continued under-utilisation of services, it would be difficult to attain the milestones contained in the national health strategic plan [5] as well as the 2030 Sustainable Development Goals in the country. To date, several demand and supply factors have been identified in quantitative research as reasons why women do not use maternal health services in Nigeria. These include ignorance and inadequate knowledge, family-related/personal factors, perceptions relating to costs and distance to services, preferences for cultural/traditional/religious forms of care, perceptions about poor quality services, and contextual factors [1, 2, 11–13].

Unfortunately, only a few of these assessments of non-use of maternal health services have specifically focused on primary health care service utilisation [14–16]. Furthermore, very few have documented the views of women in ways to enable the proper understanding of deeper reasons that women do not use skilled pregnancy care, especially at the primary health care level in rural areas. We believe that hearing the voices of rural women and men on these issues will provide a greater understanding of the related issues and lead to a more accurate design of interventions to improve women’s access to skilled pregnancy care in the country.

Reducing maternal mortality in rural areas is currently one of the most important unmet public health needs in Nigeria. It is not only a matter of equity and gender equality, but it is also one of the human rights and social justice. Therefore, the objective of this study was to investigate the reasons why women do not use PHC centres for skilled pregnancy care in Nigeria, and to make recommendations for interventions to increase access to skilled care for pregnant rural women. The study was guided by the behavioural model of health services utilisation [17, 18], and literature on demand-side barriers to health services utilisation [18, 7, 19, 20] The behavioural model proposed by Andersen and Newman identified three sets of characteristics that determine individual use of health services, namely predisposing, enabling and needs factors. The predisposing factors comprise of individual demographic and socioeconomic characteristics such as age, sex, marital status, education, occupation, and family size, and beliefs among others. The enabling factors include characteristics that influence access to health services and health personnel such as family resources, the ratio of health personnel and facilities to the population in a community, and price of health services among others. The need factors which are the most immediate determinants of health services utilisation include perceived illness or the probability of its occurrence by the individual or her family, and evaluation of the condition. This suggests that the use of a health facility can be influenced by a woman’s perception of the relative importance of modern versus traditional health care. This analysis draws mainly from enabling and needs factors. A quantitative analysis of the predisposing factors in the study location is documented elsewhere [21].

## Method

### Study design and setting

The study was part of a formative research project intended to identify elements to include in the design of an intervention for improving the access of rural women to skilled pregnancy care in rural Nigeria. An interpretive description design was used given the exploratory nature of the study, while the analysis and presentation followed the Standards for Reporting Qualitative Research (SRQR). The study was conducted in Esan South East (ESE) and Etsako East (ETE) Local Government Areas (LGA) of Edo State in southern Nigeria. Both LGAs are located in the rural areas of the state, adjacent to River Niger, with Estako East in the northern part of the Edo State part of the river, while Esan South East is in the southern part. Administratively, each LGA comprises of 10 political/health wards and there are several communities in each ward. Subsistence farming is the major source of livelihood in the communities. The two LGAs have a total population of 313,717persons, with ESE accounting for 167,721 and ETE accounting for 145,996. PHC centres are the principal source of maternity health care in the two LGAs. However, ESE LGA has one General Hospital in Ubiaja (headquarters of the LGA) while ETE has one General Hospital in Agenebode (the LGA administrative headquarters) and another in nearby Fugar City. Several private hospitals also exist in both LGAs that offer maternal and child health services of various degrees of quality and cost. These public and private facilities are used as additional to the existing PHC centres or for referral maternal health services.

A quantitative survey of 1408 women conducted in the two LGAs as part of the formative research had revealed that many of the women do not use the PHC centres for antenatal care and childbirth [21]. To explore deeper reasons why women do not use PHC centres for skilled pregnancy care, we conducted focus group discussions with various categories of women and men in various communities in the LGAs.

### Study participants and data collection procedure

A total of 20 focus group discussions (FGDs) with men and women in marital union were conducted from July 29 to August 16, 2017, to elicit their preferences, beliefs and perceptions about maternal health, and utilisation of maternal and child health care services in the primary health care facilities. Ten FGDs were held in each Local Government area. The groups were organized along the focus of sex and age to enable participants to speak freely as the presence of the opposite sex and older persons may compromise the quality and accuracy of data [22, 23]. Two groups were constituted for each of the following age categories: women less than 30 years old, women aged 31–45 years, men less than 40 years, men aged 40–54 and men aged 55 and over. The number of FGDs conducted for each age category was pre-determined based on the investigators’ knowledge of the communities. Each group consisted of 6–12 participants who were recruited by a gatekeeper through face-to-face contact. Many of the FGDs were conducted in Pidgin English and a few in the local language. The group discussions were tape recorded. The data were collected by trained field assistants and supervised by an experienced researcher. Female field assistants moderated the groups for women whereas male assistants moderated for the male groups and each group had a note-taker. The FGDs were conducted in convenient locations in the communities chosen by the participants and each discussion lasted between 60 and 90 min. A focus group discussion guide was prepared by the investigators and pre-tested in a community which has similar characteristics with the study locations. A few new questions were added while some sentences were rephrased following the pretest. The FGDs focused on where women in the communities access maternal care and the reasons why they do not use the PHC centres located in their communities. Probes included affordability, distance, and transportation among others. Ways to improve the access of pregnant women and children to health care offered by PHC facilities were also solicited in order to obtain deeper insights into the reasons that women do not use the PHC facilities for pregnancy care.

### Data analysis

The FGDs were tape-recorded and transcribed verbatim. The FGDs that were conducted in the local language and Pidgin English were transcribed by speakers of the language who are also proficient in the English language. A code list was generated by two authors from the research questions and other codes consisted of those emerging from the narratives. Computer-assisted software (Atlas.ti 6.2) was used to code and organize the codes into relevant themes. The analysis consisted of a description of the content and form following identified relevant themes. To ensure consistency and credibility of the data and the findings, member check, source, and investigator triangulation and peer review were employed. The findings were presented to selected respondents for confirmation in an intervention design meeting with the community members and other stakeholders. Thematic saturation was confirmed by the team of researchers involved in the data collection, transcription and analysis. A reasoned consensus on the emerging themes was achieved after depth discussion of the findings among the investigators.

### Ethical consideration

Ethical approval for the study was obtained from the National Health Research Ethics Committee (NHREC) of Nigeria – protocol number NHREC/01/01/2007–10/04/2017. The communities were contacted through lead contact persons, and permission to undertake the study was obtained from the Heads (Odionwere) of the communities. The participants were informed of the purpose of the study, and individual written informed consent was obtained from them. They were assured of the confidentiality of information obtained, and that such information would only be used for the study and not for other purposes. No names or specific contact information were obtained from the study participants. Only men and women that agreed to participate in the fully explained study were enlisted in the study.

## Results

The characteristics of the participants are described and the reasons for non-use of PHC centres are presented thematically with quotations showing the FGD category, and location.

### Characteristics of the study population

There were 183 participants, 106 male, and 77 females. The average age of the men was 47 years and 32 years for the women. Most (*n* = 48) of the male participants attained primary education, many (*n* = 41) had a secondary level of education and only 6 attained higher education. The majority (*n* = 43) of the female participants had primary education and 8 attained higher education. In all, 15 participants had no education (11 males and 4 females). Most of the males were farmers (*n* = 64), whereas the majority of the females were traders (n-37) and farmers (*n* = 22). The average number of living children reported by the participants was 4.9 (approximately 5 children). The male participants reported more living children than the females, the average for the men being 6 and 4 for the women. Most of the participants were Christians.

### Why rural women do not use PHC facilities for skilled pregnancy care

Responses on the reasons for non-use of primary health care facilities for pregnancy care are presented using four broad themes: physical accessibility factors, quality of care, cost of care, and other (See Table 1) with the associated quotations.

**Table 1:** Themes and number of quotations

Broad theme & number of quotations	Sub-theme
Physical accessibility factors (*n* = 38)	Bad road
	Transportation
	Distance to facility
	Facility not always open
Quality of Care (*n* = 123)	Provider competence
	Unfriendly attitude of providers
	Fear of providers and preference for Traditional Birth Attendants (TBAs)
	Inadequate providers
	Long waiting time
	Inappropriate referral
	Inadequate supply of drugs
	PHC environment/facilities
Cost of Care (*n* = 49)	Direct cost of care
	Inability to pay
	Informal payments
Other (*n* = 22)	Husband support
	Misinterpretation of signs of pregnancy complications

### Physical accessibility factors

Non-utilisation of primary health care centres for pregnancy care was associated with poor road networks, difficulty in getting transportation, long distance to a facility, and uncertainty about the facility being regularly open for patients.

#### Bad road

For women who want to use PHC centres for pregnancy care, poor road networks were a major constraint, with the result that they resort to using traditional birth attendants (TBAs) who live within the community. Bad road was a big problem particularly when the PHC cente is not located within a short trekking distance. Speaking on this barrier, one of the male participants said:

Yes, there are some old women who are mature enough to assist women to give birth, if we cannot take them to the nearest health centre because sometime this our road is very rugged, it is not motorable, it is just recently that the sun is shining [dry season] that it is motorable. For the past few months due to heavy rainfall, it has been difficult to go through the roads - so we use these women who have the skill to help us to give birth (FGD03, Men <40 years old, ETE).

In this same group, another participant stressed the impact of bad roads “You [referring to the moderator] as you were coming you saw how bad our road is, it is too bad. In the night now around 12 midnight when a woman falls into labour, what will we do? So, this woman here [referring to a TBA in his community] she can help. It is not that we do not want to go to the health centre’ (FGD03, men <40 years old, ETE). Corroborating the views of men on bad roads, a female participant submitted: “It [bad road] can lead to miscarriage since the road is not good, it becomes risky for a pregnant woman. Assuming we have it [a facility] here, it would have been easier for us. That is, it” (FGD01, women < 31 years, ESE).

#### Transportation

Difficulty in getting transportation was a major hindrance to using a PHC facility for pregnancy care, particularly for those who do not reside within a walking distance to the facilities. The primary means of transportation in these rural communities is motorcycle and they are not usually in large number. Speaking on transportation as a challenge, a male participant stated:

Like me, I have a motorbike that I can use to quickly take my wife to the health centre [PHC] during labour but for those who don't have a bike, they find it difficult to get there. Even if they have the money, to get transportation is very difficult, that is why in this situation we go to TBAs instead of staying at home to give birth to the baby. That is what we do in this community to help our women (FGD03_02, men <40 years old, ESE).

Speaking on transportation as a major hindrance, a female participant said: “… we have health centres in other places but before we will get there, we may encounter danger on the way. We don’t get a motorbike in time. We have to trek before getting transportation; the health centre is very far. It is after angle 90 the health centre is located” (women < 31 years old, ESE).

#### Distance to facility

Difficulty in getting transportation becomes a more complex barrier with long distance to a PHC centre particularly for participants who live in communities where there is no nearby PHC centre. Some of the female participants narrated how they walk long distances until they can get a motorbike to a facility. To many of them, the best thing to do is to give birth at home. Speaking about a personal experience, a male participant said:

When my woman conceived the other time, the place I took her to was very far from here. Before we got to the hospital with the pregnancy it was by the grace of God. And after everything, we lost the baby, but my wife survived. But till date she still gives birth. If a health centre was nearby when she fainted, I would have taken her to the nearby health centre on emergency. Probably I wouldn’t have lost my baby. That’s my plea, that we should have a health centre near us (FGD05, men 55+ years old, ESE).

Due to a far distance to a health facility, a male participant said they handle pregnancy care in their own way. When the moderator asked how the participant explained: “we have some leaves we give women in case of prolonged labour. When a pregnant woman uses the leaves, she will give birth” (FGD03, men < 40 years old, ETE). In ESE, a male respondent reiterated this view when he said: “Since there is no health centre and we have no money, what we use to take care of our wives are these herbs from the bush. Since it is God who created it, we pluck and bless it” (FGD03, men < 40 years old, ESE). Expressing the challenge of far distance to a facility made worse by scarce and poor means of transportation and bad road, a male participant stated:

Yes, it's far. We go through three different communities before we get to the health centre. Transportation is not available at night and the road is not in good condition. For these reasons, before you get to the health centre, the baby may get weak or the woman gets so tired and lose strength. That is why we request for a nearby place for a health centre (FGD05, men 55+ years old, ESE).

Expressing their predicament with the accessibility barriers, a female discussant said:

Yes, the reasons we don’t want to go there are many. If labour starts at midnight, the road is bad. You cannot find someone to carry you there. You are afraid not to fall from the motorcycle, you know if a pregnant woman falls from a motorcycle it is another problem. So, you will just decide to give birth at home (FGD02, women 31-45 years old, ESE).

#### Facility not always open

Sometimes, when they go through the bad road, travel the long distance, the PHC centre will not be open. Thus, uncertainty about the facility being open for patients at all time was a problem. Narrating his experience, a participant said: “I want to say something…the day my woman wants to give birth, as we got there, I saw that the door was locked, do you understand? They locked the gate. We did not see anybody to assist us. Then, I rushed my wife home and my wife gave birth in my house” (FGD03, men < 40 years old, ETE). In another FGD for women aged 31–45 years old in ETE, a participant said they usually start work at 10 am and close at 2.00 pm”. A male participant in ESE observed that “Sometimes the nurses abandon the health centre and go to the market and other places” (FGD04, men 40–54 years old, ESE). It was also observed by some participants that a reason why some facilities are not always open is that the providers are not resident in the community. Some of the providers come from a far distance due to lack of accommodation at the PHC facility for them. In ETE, a participant narrated what she said was their usual experience: “Because we do not have a hospital here if you are pregnant, we suffer. We do not see anyone to treat us. If we get to Okpekpe or Ebele [other nearby communities with a PHC facility]; we may not see any provider to treat us there. If we want to give birth, we suffer here before we give birth (FGD02, women 31–45 years old, ETE).

### Quality of care

Narrating from personal and the experience of women known to them, the participants pointed to various aspects of quality of care such as provider competence, negligent and unfriendly attitudes of PHC providers, physical environment and facilities, inadequate supply of drugs, inadequate providers, long waiting time and inappropriate referrals as reasons that discourage them from using the facilities for pregnancy care. To many of the women who mentioned the quality of care as a barrier, they would rather give birth at home than go to a PHC facility. Expressing this view, a woman said:

Not that we prefer TBAs, but when you go there [PHC centre], no help, they will leave the woman to suffer before she gives birth after she has given birth, they will come and carry the baby, bath and pour powder on the baby. Would they say they assisted the woman's childbirth? What is the need, instead of that, you stay in your house because that suffering you would suffer in the house, you will still suffer it there [PHC centre], because of that you better stay at home, you do everything, God will still help you. What’s the need now, and the money they would have collected for nothing sake, you better use that money to prepare your soup and do everything for your child (FGD02, women 31-45 years old, ETE).

#### Provider competence

Speaking from her experience, a woman said:

Like the time I gave birth, I was in labour, by the time I was pushing for the baby to come out I did not see the nurses again. I started shouting on them to come and help that the baby is coming out and if my baby touches the ground, I will not be happy with them. When they heard that, they came and started telling me that they are sorry, with that kind of experience I don’t think I will go there next time (FGD01, women <31 years old, ESE).

Another woman was of the view that giving birth in a PHC facility would have led to death for her and her baby. She said: “To me I will not go to the health centre [PHC] to give birth because this my last twins, I gave birth to them at the private hospital and I know how the nurses and doctor took good care of me, if it was health centre my baby and I will not survive (FGD01, women <31 years old, ESE).

In the FGDs conducted with men, some of them were of the opinion that the use of PHC facilities for pregnancy care is related to the competence and dedication of the providers. One of the participants said:

Yes, in those days, the health centre was the best but now you hardly see good health workers, but when you go to the private hospital, they will give you good service. Even when you take a pregnant woman to the health centre [PHC] they will be reluctant to treat. Please, you people should also talk to them to work hard and do their best (FGD03, men <40 years old, ESE).

Another male participant stated: “The time when that nurse [referring to a particular nurse] was here, people were using the facility very well. One woman gave birth to a triplet there when that nurse was there. Anytime we just knock the gate she opened, the nurse was staying there. It pained everybody when she was transferred to Okpella” (FGD03, men <40 years old, ETE). Expressing this same view, a man in ESE said:

There's a woman who was here but now transferred. When she was here, there were no complains. In short, my daughter gave birth to twins there when she was here. But today, people complain bitterly. So, the few women that still attend the health centre are just those that are new in this community. Those who understand how they operate in this health centre do not go there anymore (FGD04, men 40-54 years old, ESE).

Inability to recognise the signs of labour by the providers was another reason. A participant stated: “the reason why we do not go there [PHC centre] is that when you get there, they do not take good care of us, sometimes when you are clearly in labour, they will say you are not yet in labour and they will just leave you alone inside the labour room” (FGD01, women < 31 years old, ETE). Expressing his view, a participant alluded to provider incompetence and arrogance as a barrier to utilization of PHC for pregnancy care in the rural communities. He said:: “I don’t like the way they attend to people. They don’t treat people well. We don’t have the voice to talk to them, whatever you say, they work for the government and you cannot petition them to anybody. They do whatever they like” (FGD04, men 40–54 years old, ESE).

#### Unfriendly attitude of providers

The unfriendly attitude of providers was an aspect of quality of care that reoccurred in many groups as a major reason why women avoid PHC. Those who would have made effort to go through the barriers of bad road, long distance and difficult transportation are discouraged to seek skilled care in PHC centres by the unfriendly attitude of nurses. Emphasizing this problem, a female participant said:

Because the health centres do not take good care and the road is too far. More so some health workers do not give good care. The nurses do not behave well; having known that a woman under labour can be angry because of the pains, instead of the nurses taking things easy with the women they will worsen everything (FGD02, women 31-45 years old, ESE).

Another woman submitted: “To me, I can’t use the health centre because the suffering and insult is too much. I can’t allow a small nurse to insult me on my pregnancy” (FGD01, women < 31 years old, ESE).

#### Fear of providers and preference for home/TBAs

In some cases, the respondents would not use a PHC centre for pregnancy care because they feared to go to a hospital, feared nurses and preferred traditional birth attendants who they claimed were friendlier. Expressing the reason for the fear of the hospital, a participant said:

“Some will blame you when there is bleeding and loss of strength. They will say that's why nurses advise you to be eating vegetables. When women come to the health centre late, doctors will say that they are late. When there is a low blood level in a woman that has not visited the clinic since they will blame the woman for the low blood level. That is why we are afraid because there are some hospitals, they are just there, they don’t know how to care for people. When a woman under labour comes to them, they will say no blood; she is weak and so on” (FGD02, women 31-45 years old, ESE).

Two participants in a group in ETE had all their children at home because of fear of nurses. One of them said: “I did not go there because of fear” when the moderator probed further, she continued: “yes, I fear them, nurses do not hold someone in labour, they will be abusing you, that is why I did not go there”. Another woman in this group added “they will just be shouting, shouting, “did I send you? Did I impregnate you?” (FGD02, women 31–45 years old, ETE). Narrating an experience, another woman said:

There was a day I took my baby for immunisation, I saw a woman who they said has been there for the past two days and all the nurses on duty were shouting at her instead of petting her. When I saw that kind of a situation, I was afraid to go and register there because I know they will do it to me when the time comes, but when you go to a private hospital they will pet and pamper you, that is why we go to private hospitals. In the health centre their shouting can make someone lose her baby (FGD01, women <31 years old, ESE).

Expressing a preference for TBA, a female participant submitted that “The native medicine is as good as maternity. And it is only God who can help someone wherever you give birth. I have given birth before in the traditional health home. It was very good for me” (FGD02, women 31–45 years old, ESE). Speaking on her preference for TBAs, another woman said:

Like my sister who gave birth there [PHC centre] when she got there the nurse was shouting on her to push, that when she was doing it if it was not sweet and now, she is shouting. So, with that experience, I choose to always go for TBA and each time I am there and I complain the woman will attend to me fast and give me herbs to drink, also pet me, before you know it the baby has come out. So, to me, that is why I prefer TBA (FGD01, women <31 years old, ESE).

#### Inadequate providers

Some participants do not use PHC because the providers are few, not always available and it is hard to meet a doctor in the facilities. Expressing this view, a male respondent said:

The major problem I know we have is that we have only one nurse and I think it is good for us to have a specialist, then if it is difficult for them to bring a doctor, they should increase the number of the nurses. At times if the nurse is not around, this PHC centre will be completely closed down. Sometimes if women are in labour they will not meet the nurse and we have to take her to Bode, which is 12km away. They can send another nurse or increase them up to three so that this PHC will function very well (FGD05, men 55+ years old, ETE).

For many people, the touch of a doctor increases satisfaction with the health care received. Thus, the absence of doctors in PHC facilities makes the participants think the quality of care is low. One participant stated: “before you see the doctor, the woman would have suffered, you suffer before you see the doctor” (FGD02, women 31–45 years old, ETE). There are PHC facilities without a midwife. Speaking on this, a participant stated: “I cannot lie to you, there is no doctor, no nurse here o…. It is the woman’s [TBA] place they go for pregnancy care and childbirth. One woman gave birth in the woman’s place last week. No nurse in our health centre here” (FGD03, men < 40 years old, ETE). Speaking on providers not always available, another participant added: “What my people said is the truth, sometimes if a woman wants to give birth, she may not meet a nurse because the nurse is not always around on weekends and sometimes in the late hour. So, we need more nurses to assist and a doctor” (FGD05, men 55+ years old, ETE). Responding to the view about money as a deterrent, most participants in a group for women aged 31–45 years old responded: “no money at all (general response) it is doctors and nurses that we don’t see, that’s why we don’t want to go there” (FGD01, women 31–45 years old, ESE). Narrating her personal experience, a participant said: “Sometimes, when you get there you won’t see nurses. Like when I gave birth to my second child before the nurses came, I have already given birth at the door (entrance). We hardly see the nurses” (FGD01, women < 31 years old, ESE).

#### Long waiting time

For some of the participants, the time they wait before seeing a provider in a PHC facility deters them from using PHC for pregnancy care. The participants attributed the long waiting time to the uncaring attitude of the providers, long procedures in registration even during an emergency, lack of doctors and necessary drugs in the facility. Sometimes, women are sent to meet a doctor somewhere or to purchase drugs before the PHC provider attends to them. One of the men expressed his dissatisfaction with the waiting: “The one we have here, why I said I don’t like it [PHC] is because they are not attentive. When they ask you to come, if you get there before they will attend to you it will take a long time. And even if you call them since they live in the same building before they will come out, it will take a long time, and you have to shout. So, their delay is so much” (FGD04, men 40–54 years old, ESE). For a female participant, the PHC providers are trying to provide good service but the waiting time is long. She said: “they are trying but before they will look after you, they will first waste your time, shout on you, insult you before they will eventually take care of you...” (FGD01, women < 31 years old, ESE).

#### Inappropriate referral

Another reason why some women do not use PHC facilities is inappropriate referral to private facilities owned by the matrons who work in the PHC facilities. Speaking on this, a participant said:

“We are saying health centre, can you imagine that when you get to the health centre the matron there will refer you to her own private clinic and tell you to pay a certain amount and disqualify all the baby things you bought and ask you to buy them again from her clinic. You that register in the health centre they will end up not attending to you well, that is the reasons most women go to the herbalist houses or use TBA and drink all the concussion that later affect the woman or the baby which is not supposed to be so” (FGD01, women <31 years old, ESE).

#### Inadequate supply of drugs

Another dimension of quality of care that was prominent in the narratives was the non-availability of drugs in the PHC centres. Many respondents claimed that drugs are hardly available in the PHC centres and when available, the drugs are sold at rates higher than the conventional price. Some of the participants reported that they are forced to buy the drugs at a higher price because sometimes the nurse will insist that they buy the drugs before service delivery. Speaking on non-availability of drugs, a participant said: “in the health centre [PHC] they don’t give drugs, it is only to take a child there and give BCG [Bacille Calmette-Guerin] that is the only thing they do there” (FGD01, women < 31 years old, ESE). Reacting to the opinion of a participant on drug supply, a male participant said:

I think what he just said is very important, no drugs here because sometimes after prescription they will say we should go to other places to buy. If all these things are available here to buy them, it will be easy. Before we transport from here to Agenebode to buy those drugs, even if the drugs are expensive here, we will still buy. I think is better to have drugs here because we call here a PHC centre - a PHC centre without drugs cannot operate; provision of drugs is the first thing the government should do, thank you (FGD05, men 55+ years old, ETE).

#### PHC environment/facilities

Another quality of care barrier that was commonly raised by the participants is the PHC environment and the lack of necessary facilities. Describing the PHC facility in his community, a male participant said: “… No room for the nurse to stay, no toilet, no water, no fence, no gate, no security man that is supposed to call the nurse if there is an emergency and no telephone for communication in the PHC centre” (FGD05, men 55+ years old, ETE). Speaking on this, another participant stated:

What I think of these issues! like this our [PHC centre] why we don't normally go there again, the problem we have, if a woman falls into labour at night, you cannot say let me rush to the health centre we don't even have light, no generator, everywhere is bush, that is the problem we always have for that primary healthcare centre, everywhere looks dirty and no light, you can't even say you are in labour in the night and you rush there, that is the problem we always have there (FGD01, women <31 years old, ETE).

## Cost of care

### Direct cost of care

The direct cost of care in the primary health care centres was expressed by women and men as a barrier, although they claimed it is cheaper than the cost of care in the private and specialists’ hospitals. Speaking on the cost of care as a reason for non-utilisation of primary health care facility in his community, a man said: “Why our women are not using this PHC centre is because the price is high and it is a government hospital, when our women give birth here, they are charged Naira10,000 (US$27.8), Naira12000 (US$33.3), Naira8000 (US$22.2). But if we go to Bode hospital [secondary facility] we will pay the transport, but it will be cheaper, that is why they don’t come to this PHC” (FGD04, men 40–54 years old, ETE). In another FGD, a man who is a community leader averred:

I am a chief in this community, the reason why this PHC centre is not moving well is that the price is high [high cost of care]. They brought this PHC centre to us so that it will be easier for us but the general hospital [secondary facility] is cheaper. If they [PHC] assist a woman to give birth or give drugs they charge … this and that. Any small sickness they will charge Naira3000 (US$8.3), Naira4000 (US$11.1) but in general hospital, it is Naira 500 (US$1.4), 1000 (US$2.8). This PHC centre is now worst (in terms of costs), that is why it is not moving well, so report to them so that they will reduce the price. Because there is no documentation of it, they charge anyhow (FGD05, men 55+ years old, ETE).

Affirming the high cost of care as a reason for non-use of PHC, another participant doubted that the government approved the high charges:

“... for example, my child gave birth to a daughter, I paid Naira50,000 (US$138.9) in the maternity [PHC]. I told you about the woman that's not serious with her job. I thank God the mother and the child are alive. But I'm not sure the government would charge women such amount for giving birth in a Maternity when there were no complications” (FGD04, men 40-54 years old, ESE).

In the FGDs with younger men in ETE, there were differences in opinion about direct cost care; whereas some of the participants were of the view that the charges are high, others said it is not too high. One of those who said it is high stated: “when my woman gave birth there, they charged us about Naira7,000 (US$9.4)” (FGD03, men < 40 years old, ETE). Some of those who said the cost of care is high attributed it to their inability to pay. Also, some younger women (< 31 years old) said the direct charges are not too high. In contrast, older women perceived the cost of care as high and discriminatory. One of them declared: “Yes, they charge, if you give birth there, they charge Naira8,000(US$22.2), Naira6,000(US$16.7), Naira7,000(US$9.4). That is how they do it. If you give birth to a male child, you are charged Naira8,000 (US$22.2), if you give birth to a female, you are charged Naira5,000 (US$13.8), Naira6,000 (US$16.7)”. Another participant in the same group added: “if you give birth to twins instead of them to be happy that you had twins, they will charge you more” (FGD02, women 31–45 years old, ETE). Comparing the direct cost in PHC facilities to the cost of getting birth assistance from elderly women or TBAs, one of the participants said: “anything you want, you give the person, whether it is Naira500(US$1.4) or Naira1,000(US$2.8), you give the person, that is how we do it (FGD02, women 31–45 years old, ETE).

Apart from the direct cost of intrapartum care, the women also complained about the direct cost of antenatal care. One of them in FGD02 for women aged 31–45 years in ETE said: “Each time you go for a check-up you pay 500 Naira” another participant interjected: every two-two weeks [bi-weekly]. In an FGD with women aged < 31 years in ESE, the women stated that registration for first timers is Naira300–700(US$0.8–1.9), every antenatal care visit is Naira200–300 (US$0.6–0.8), but the cost of intrapartum care varied between Naira5,000 to 15,000 (US$13.9–41.7). This varied experience by women in the same State seems to confirm the claim of a male discussant that the amount one pays is negotiable: “Yes, as we all know, all fingers are not the same. When my wife gave birth, I know how much I spent. They all vary. Depending on how you explain yourself to them [the providers at the PHC centre], they will help you...” (FGD05, men 55+ years old, ESE).

Some male participants did not consider the direct cost of care a problem. One of them said: “when a woman is pregnant and she goes for antenatal, it is the husband’s responsibility to pay the bill. The amount on the bill is not compared to the life of pregnant women. So, the bill is sorted out without fear or worries of its cost” (FGD04, men 40–54 years old, ESE). Another participant in this group concurred: “We should all know that as a man that already has a wife, and it is time for childbirth, any amount you are to pay is good you pay it. Your concern should be that the woman and the child survive. Is that not right?” In response, some of the participants agreed with him and others disagreed with him. In another FGD with men aged 40–54 years, the direct cost was viewed as a problem and they considered that maternal health care services should be free (FGD04, men 40–54 years old, ESE).

In spite of the high cost, some women were of the view that the cost of care for maternal health is a lesser barrier than the availability and attitude of the providers. Speaking on why they do not use PHC facility, a participant said: “It is not because of money. If you are holding money and you cannot find what to buy, that’s a problem. It is said that when there is money, you don’t mind the cost of what you buy. If you are holding money, and you are looking for doctors and nurses, that’s a problem on its own...” (FGD02, women 31-45 years old, ESE). Other participants in her group agreed with this view. In another group, a younger woman expressed: “The cost is too much but the worst aspect is that there is no adequate care for the pregnant women; they don’t pet rather they shout at us” (FGD01, women < 31 years old, ESE).

#### Inability to pay

Closely associated with the direct cost of care is the inability to pay. Asked his opinion about the direct cost of care as a reason for non-utilisation of PHC centres for pregnancy care, a man responded: “No, not because of high cost, it is sometimes due to inadequate funds” (FGD03, men < 40 years old, ETE). Expressing this further, another participant stated:

You see, if you take a view of this community as a whole, we have no industry, factory or something. We are all farmers and farmers depend on annual harvest ... Now, for a farmer to have even Naira50(US$0.1) in his pocket is rare not to talk of the other children how to feed them, and you thinking of transport to a PHC centre. So, unless you go into borrowing from meeting [contributory fund] before you can get money to use a PHC centre (FGD03, men <40 years old, ETE).

Expressing the fact of lack of money, a participant narrated what happened to a woman known to her: “there is a woman here; she was using a clinic at Okpekpe. They gave her time to come. When the time came, she had no money to go until the time for her to give birth. The woman became sick and she died with the baby in her womb” (FGD02, women 31–45 years old, ETE). In ESE, lack of money was said to be one of the reasons why they resort to using herbs for pregnancy care.

#### Informal payments

Another barrier to utilisation of PHC for pregnancy care is demand for materials which the participants consider unofficial. One of the participants said: “another one is that when you get there, they ask you to buy disinfectant, buy a towel, buy everything and when you buy them, they will not allow you to take them back again” (FGD02, women 31–45 years old, ETE). When the moderator asked if those items are not used for their birth care, a participant responded: “they use some, but they do not use all, they keep the remaining as their own”. Other participants in this group added that they also ask for kerosene. A participant explained why they demand for kerosene: “if the woman finish giving birth to her baby, they will put the kerosene into a kerosene stove to boil tea and give the woman to drink, but they will not return the remaining kerosene to the woman, they will keep it at the health centre for their use” (FGD02, women 31–45 years old, ETE).

## Other

### Husband support

Support of husbands in pregnancy care was extensively discussed in the FGDs with men and women. Most men, as well as women, claimed that husbands support in diverse ways in pregnancy care. However, from the narratives, it was obvious that minimal or no support from husbands in forms of money, assistance in domestic chores, during pregnancy hindered some of the women from using the available services in the PHC facilities. Speaking on this, a participant said: “In many aspects, but our men always complain about our clinic because not everybody has money ...” (FGD01, women <31 years old, ETE). Another participant submitted: “what if they [husbands] do not have money, and they tell you that they do not have, what will you do?” In the same group, another woman added “some [men] run away” (FGD02, women 31–45 years old, ETE). Expressing his view on husbands’ support, a male participant said:

Yes, it is true we give them money to go to the clinic. However, all men don't give their wives money anyway. We understand that poverty is the cause of such. In some homes, the men find it difficult to raise money for their wives to go for maternal care...But because of poverty, some men fail in allowing their wives to go for antenatal care. Some other times they give their wives money that won’t even be enough... (FGD04, men 40-54 years old, ESE).

Some women receive little or no support from their husband. As a result, they forgo skilled care because they cannot afford it due to other competing needs such as care of other children. Expressing this, a female participant said:

The men we have in this village, we just know that we are giving births. Not that we really need the pregnancies. Because a man who really needs a baby, when the wife is pregnant, there are certain works she can do, and there are certain works you cannot do. On the day of clinic, when you tell the man today is clinic day, it will be difficult for him to bring out that money from his pocket. Baby things, he won't bring money out to buy. It is only the woman who goes to the farm, go everywhere struggling to look for all those things to buy baby things and how to take care of the baby. These are the problems we have in this village (FGD02, women 31-45 years old, ESE).

#### Misinterpretation of signs of pregnancy complications

Some of the women do not use primary health care centres because they interpret a lack of signs of complications and ill-health in pregnancy as indications that they can give birth at home without problems. One of the participants who gave birth at home said: ‘the reason why I do not go to Okpekpe [referring to the location of the PHC centre] is because when my due date is near, I will try my best and God will help me to give birth in the house’ (FGD02, women 31–45 years old, ETE).

## Discussion

The study was designed to explore reasons that women in rural communities in Edo State, Nigeria, do not use skilled pregnancy care offered in primary health centres. Previous studies conducted in different rural communities in the country have reported substantial under-utilisation of orthodox health facilities by women for pregnancy care [2, 4]. Our recent quantitative study in the same communities where this qualitative study was conducted showed that less than 47% of pregnant women gave birth in PHC centres, while 25% gave birth at home even when PHC facilities are presumably located in districts close to where the women live [21]. Although specific associations were identified in the cross-sectional survey between various independent variables and women’s likelihood to use skilled pregnancy care in PHC centres, we decided it was important to hear the voices of women and their partners through qualitative research to identify reasons that primary healthcare facilities may not be used for skilled pregnancy care. We believe this approach is important to provide a deeper understanding of the nature of the problem.

The results of this study identified diverse reasons with detailed explanations as to why women do not use PHC facilities for pregnancy care in the two LGAs. The reasons proffered can be broadly categorized into 1) accessibility factors – poor roads, difficulty with transportation, long distances to PHC facilities and that the PHC centres are not always open; 2) perceptions relating to poor quality of care in PHC centres, including inadequate drugs and consumables, abusive care by health providers, providers not in sufficient numbers and not always available in the facilities, long waiting times, and inappropriate referrals; and 3) high costs of services, which include the inability to pay for services even when costs are not excessive, and the introduction of informal payments by staff.

Some of these reasons have been identified in several studies in many parts of Nigeria and other parts of Africa [14–16, 24, 25] for women’s under-utilisation of maternal health services. However, a specific strength of this study is the targeting of primary healthcare, women, and men in rural communities and its proficiency in investigating what women are willing to accept or not to accept in matters relating to access to skilled pregnancy care at the primary healthcare level. Many studies in developing countries show the critical role of husbands in decisions about maternal care [26–28]. Thus, hearing the women, as well as the men on the barriers to utilisation of PHC, was significant for the robust design of sustainable interventions.

While the cost of services was an important consideration for women and their partner in reaching decisions to seek pregnancy care in PHC centres, it was clear that if services are of better quality (with better availability of skilled providers and facilities), and with the facilities being more easily accessible, women will be ready to pay the costs of services. Contrary to our expectations, women’s lack of knowledge about the need for skilled pregnancy care and cultural or normative preferences did not feature as reasons for non-use of PHC for skilled pregnancy care.

In consequence of these findings, it is evident that improving the quality of care in PHC centres, addressing physical access to health facilities and cost of care and encouraging male support would be most impactful interventions for increasing women’s access to skilled pregnancy care in rural communities. However, increasing the numbers of PHC centres to improve physical access may not be an easy solution, since Nigeria currently has up to 30,000 PHC centres, which is more than adequate to serve the country’s population of 180 million people. Despite this large number, a sizeable proportion, especially in some of the most hard-to-reach rural communities in the country still do not have PHC centres, because of poor distribution, bad roads, and poor terrains. Among existing PHC centres, the majority are dysfunctional, poorly staffed and poorly equipped. Therefore, the current policy of the Federal and some State Governments to refurbish existing PHC centres [6] is recommended and would be one way to improve the infrastructure of PHC centres. Aside from this, efforts should be made to provide an adequate number of health providers in the PHC centres, to re-train providers to offer respectful and effective care and to sustainably equip and re-stock the facilities with essential drugs and consumables. This will strengthen the current efforts in the country to discourage women from using unskilled traditional birth attendants, a major determinant of high maternal mortality in Nigeria [29].

Improving roads and transportation systems is currently a major developmental challenge in Nigeria, especially in rural communities. With bad roads and poor transportation systems, the health sector has continued to bear a huge consequence by delimiting the use of available health facilities, especially in rural communities. While efforts should be made to improve roads and transportation systems in the country on the long term, short term and creative measures should be put in place to ensure that women are able to get the transportation they require to access skilled pregnancy care. This normally would include emergency ambulances provided by governments as a social safety net, as has been done in poor rural communities in other parts of Africa [30, 31].

. However, should this not be readily available, alternative and creative methods of transportation should be devised to address this important bottleneck. If such innovations and creativities are managed by the local communities with community savings and ownership, and possibly in collaboration with the private sector, it stands a huge chance of success at least on the short term.

## Limitations

An important limitation of this study is that the views and how they were expressed by participants may have been influenced by the presence of others in the group. However, specific efforts were made to organize groups by sex and close age groups, while the moderators were trained to organize the group interviews in a value-free and equal manner. Some of the participants narrated their personal experiences, but a common limitation of focus groups is the inability to generate in-depth information on individual perceptions and experiences [31, 32].

## Conclusion

The results of this study have implications for health systems reforms for the prevention of maternal mortality in the country. Low skilled birth attendance, especially in rural communities, is currently the most important challenge facing Nigeria in efforts to reduce the currently high rate of maternal deaths and neonatal mortality. The use of PHC offers the best opportunity to increase the access of rural women to skilled pregnancy care. However, the evidence from this study suggests that rural women will not use PHC for pregnancy care if they cannot physically access the health facilities, if the PHC centres offer low quality and non-respectful care if the cost is not affordable and partner support is lacking or minimal. Clearly, policies and programmes based on the revitalization of PHC through adequate budgetary allocations and good development planning that target these barriers are critical if the country desires to improve women’s access to skilled pregnancy care and reduce the number of maternal deaths.

## Data Availability

The dataset used and analyzed during the current study is available from the corresponding author on reasonable request.

## Abbreviations

BCG: Bacille Calmette-Guerin
ESE: Esan South East
ETE: Etsako East
FGD: Focus Group Discussion
LGA: Local Government Area
NHREC: National Health Research Ethics Committee
PHC: Primary Health Care
SRQR: Standards for Reporting Qualitative Research
TBA: Traditional Birth Attendants
